# Special Issue “Skeletal Muscle Adaptations to Oxidative Stress”

**DOI:** 10.3390/ijms26199809

**Published:** 2025-10-09

**Authors:** Guglielmo Duranti

**Affiliations:** Laboratory of Biochemistry and Molecular Biology, Department of Movement, Human and Health Sciences, Università degli Studi di Roma “Foro Italico”, Piazza Lauro de Bosis, 6, 00135 Rome, Italy; guglielmo.duranti@uniroma4.it; Tel.: +39-06-36733479

Skeletal muscle constitutes approximately 40% of total adult body weight, and its health is essential for overall well-being. Muscles are responsible for movement, maintaining posture, supporting bones, regulating energy metabolism, and protecting the body, all of which are fundamental functions for the individual.

However, due to the specific type of activity it performs, skeletal muscle is consistently subjected to mechanical and oxidative stress during activities. While normal contractile activity induces physiological adaptations that enable the muscle to mitigate oxidative damage, intense activity/exercise can lead to detrimental effects at both the cellular and functional levels.

In fact, excessive muscle stretching and intense physical exercise can cause rupture of myofibrillar filaments, resulting in a loss of muscle function due to disruptions in the excitation–contraction coupling mechanism. These events trigger an inflammatory response and increase the production of reactive oxygen species (ROS). ROS are continuously generated in the body, and under normal conditions, they are rapidly neutralized by cellular enzymatic and non-enzymatic antioxidant defenses. In skeletal muscle, low concentrations of ROS are involved in modulating cellular signaling pathways and are essential for normal force generation. However, elevated ROS levels can cause irreversible modifications to cellular structure such as DNA, lipids, proteins, and carbohydrates, which may impair cellular function and reduce force production, thereby contributing to muscle fatigue with the compromise of contractile activity [1,2,3,4,5].

Consequently, understanding exercise-induced muscular adaptations at the molecular and biochemical levels, as well as their influence on cellular signaling pathways, is crucial for developing training protocols that promote individual health.

In recent years, there has been a significant increase in the number of scientific publications addressing muscle physiology and pathophysiology [6].

This surge is not unexpected, given that muscle tissue plays a pivotal role, as previously mentioned, in maintaining overall well-being through its involvement in posture, movement, and metabolic processes. Over the past two decades, the volume of studies in this area has become more pronounced. In particular, there has been a growing focus on investigating ROS production and its contribution to muscle pathophysiology [7].

In this context, this Special Issue, titled “*Skeletal Muscle Adaptations to Oxidative Stress*”, published in the *International Journal of Molecular Sciences*, aims to collect new research in the field through five contributions: three original research articles and two reviews. This research offers new insights into the impact of oxidative stress on muscle function and adaptation in both normal and pathological states.

There is a continuous and growing demand for strategies and/or supplements that can alleviate the negative effects of ROS produced by intense and vigorous physical activity at a systemic and general level [8].

Significant interest has been developing for motor and exercise protocols that can induce positive adaptations in the muscle, thereby improving its response to oxidative stress. This has proven particularly important in the presence of pathological conditions and/or health-related conditions associated with ROS production [9,10,11]. In this context, great interest is developing in motor protocols applied to oncology patients (Figure 1).

This same trend is found when the words “exercise therapy” or “exercise as a therapy” are combined with the word “cancer” [12].

Of particular interest are the molecular modulations that exercise induces in muscle cells and how these then translate into beneficial signals for the organism as a whole [13,14]. This is particularly relevant for cancer patients who must also undergo specific therapies that often cause systemic oxidative stress [15].

Breast cancer is one of the most prevalent malignancies worldwide, characterized by uncontrolled cell growth in breast tissue. The development and progression of breast cancer are influenced by a variety of genetic, environmental, and lifestyle factors. One critical factor contributing to tumorigenesis is oxidative stress, which results from an imbalance between the production of reactive oxygen species (ROS) and the body’s antioxidant defense mechanisms. In these patients, oxidative stress has been implicated in processes such as angiogenesis, inflammation, and resistance to apoptosis by influencing the responsiveness of breast cancer cells to chemotherapy and radiation, all of which contribute to the malignant phenotype. Therefore, the modulation of oxidative stress pathways is emerging as a potential therapeutic strategy for improving breast cancer treatment and preventing disease recurrence.

Parisi A., Dimauro I., and Moulton C. proposed a relationship between physical activity and improved quality of life in breast cancer patients undergoing drug treatment [16,17].

Moulton et al. later proposed a motor protocol for this Special Issue, demonstrating that a 16-week online supervised physical activity (PA) intervention improved cardiovascular fitness and modulated the ELOVL2 epigenetic clock assessed by DNA methylation analysis in breast cancer (BC) survivors undergoing medical treatment [18]. The reversal of the ELOVL2 epigenetic clock indicates a significant decrease in biological age within the cohort of BC patients and underscores the capacity of exercise interventions to mitigate and reverse accelerated aging processes, particularly in this vulnerable population. Moreover, improved hand grip strength and cardiovascular fitness, associated with decelerated biological age, contribute to enhanced overall health, potentially leading to a faster and more robust recovery process in these patients [18]. The authors also investigated the redox signaling induced by this protocol and identified a novel molecular vehicle for redox regulation in extracellular vesicles [19,20].

This new line of research extends the potential of studying exercise-induced epigenetic regulation beyond traditional applications [13,21,22], including in the field of oncology.

Another pathology characterized by the presence of reactive oxygen species is Duchenne muscular dystrophy (DMD) [23,24].

DMD is a severe X-linked genetic disorder characterized by progressive skeletal muscle degeneration and weakness. It is caused by mutations in the dystrophin gene, leading to the absence or dysfunction of the dystrophin protein, which plays a crucial role in maintaining muscle cell integrity. In DMD, oxidative stress is a key pathological feature, as elevated levels of reactive oxygen species (ROS) are generated during muscle contraction, exacerbating cellular injury. Moreover, the impaired antioxidant defense mechanisms in DMD further amplify oxidative damage, leading to muscle fiber degeneration, inflammation, and fibrosis, which are hallmark features of the disease.

Cernisova et al., in a fibrotic animal model of DMD, demonstrated that gene addition therapy, based on the adeno-associated viral (AAV) vector-mediated delivery of microdystrophin transgenes, significantly improves body-wide muscle function. This is associated with the protection of the hindlimb muscle from contraction-induced damage and the prevention of fibrosis deposition in the diaphragm muscle. Although this study was conducted on mice, the results are certainly promising, as demonstrated by the normalization of the diaphragm mass and fiber diameter with gene addition therapy. Moreover, a reduction in centrally nucleated fibers, collagen VI deposition, and mRNA expression of fibrosis and inflammation-related genes was observed. All these modulations are followed by an improvement in the physical abilities of mice [25].

Instead, evaluating the metabolic changes that occur in the tissue, muscle cell activity significantly influences glucose homeostasis by enhancing its uptake. During exercise, muscle contraction stimulates several signaling pathways that increase glucose transporter (GLUT) protein expression, particularly GLUT4, which is responsible for insulin-independent glucose transport. This enhanced glucose uptake allows for more efficient utilization of glucose as an energy source, particularly in skeletal muscles, where glucose is rapidly metabolized to produce ATP. Additionally, exercise improves insulin sensitivity, further contributing to increased glucose uptake even in the absence of high insulin levels. In this context, the acute and chronic effects of exercise on glucose uptake are crucial for maintaining glucose balance, thus preventing metabolic disorders, such as type 2 diabetes. Consequently, accurately tracking the dynamics of glucose utilization is essential to follow the molecular mechanisms induced by various types of exercise [26].

To this end, Fernández-Puente and colleagues developed and validated a methodology based on the fluorescence glucose analog 6-NBDG, combined with quantitative fluorescence microscopy image analysis, to assess glucose uptake in two skeletal muscle cell models: mouse C2C12 myotubes and single fibers isolated from skeletal muscle. The novelty of this technique lies in its ability to track glucose uptake in living cells, observed under a microscope, while maintaining the physiological medium without depleting any component (e.g., glucose) over an extended period. This approach is particularly important to better understand the effects of various treatments, with a specific focus on glucose uptake and metabolism [27].

The Special Issue “Skeletal Muscle Adaptations to Oxidative Stress” contains two review articles. The first is entitled “Effects of Hydrostatic Pressure on Muscle Contraction: A Look Back on Some Experimental Findings” by K. W. Ranatunga and M. A. Geeves [28].

Hydrostatic pressure plays a significant role in muscle contraction, particularly in the context of physiological and pathological conditions. Muscle contraction is primarily driven by the interaction between actin and myosin filaments, regulated by the excitation-contraction coupling mechanism. However, external factors such as hydrostatic pressure can influence the mechanical properties of muscle tissue. Increased hydrostatic pressure, which occurs in tissues exposed to elevated fluid pressures, can alter the muscle’s ability to generate force by modifying the structural integrity of muscle fibers and disrupting normal ion gradients across the sarcolemma. In aquatic environments or clinical conditions such as edema or deep-sea diving, elevated external pressure can affect the muscle’s responsiveness to neural stimulation, potentially impairing muscle function. Conversely, the application of controlled hydrostatic pressure can be utilized therapeutically to enhance blood circulation and promote muscle recovery, as it can reduce swelling and improve nutrient delivery to muscle tissues. Hence, understanding the relationship between hydrostatic pressure and muscle contraction is crucial for advancing treatments for muscle disorders and optimizing physical rehabilitation strategies. In this review, the authors re-examine the basic findings in pressure perturbation studies conducted on muscle fibers, emphasizing aspects that are not fully understood in this topic and then highlight some ideas for future research [28].

The second review paper is entitled “Advanced Cellular Models for Rare Disease Study: Exploring Neural, Muscle and Skeletal Organoids” by Bombieri and collaborators [29].

Organoids, three-dimensional cellular structures that replicate the architecture and function of native tissues, have emerged as powerful tools for studying muscle physiopathology [30]. In the context of skeletal muscle, organoids derived from pluripotent stem cells or primary muscle cells form a model system that mimics the complexity of muscle tissue, including the interaction between different cell types such as myocytes, fibroblasts, and endothelial cells. These muscle-derived organoids can be used to investigate a variety of muscular diseases, including Duchenne muscular dystrophy (DMD) and other genetic myopathies, by allowing for in vitro modeling of disease progression, cellular degeneration, and regeneration. Organoids also provide a platform for studying the effects of oxidative stress, inflammation, and nutrient signaling on muscle function and repair. Furthermore, their use in drug screening and therapeutic testing is increasingly valuable for identifying potential treatments for muscle-related disorders, as they offer a more physiologically relevant environment compared to traditional two-dimensional cell cultures. The application of organoid technology to muscle physiopathology is revolutionizing our understanding of muscle biology and holds promise for the development of targeted therapeutic strategies [29,30].

Induced pluripotent stem cells (iPSCs) have revolutionized the study of muscle physiopathology by providing a versatile and patient-specific model to investigate the molecular mechanisms underlying muscle diseases [31,32]. iPSCs are generated by reprogramming somatic cells in a pluripotent state, allowing them to differentiate into various cell types, including myocytes. This ability to generate muscle cells from patients with specific genetic mutations, such as those seen in Duchenne muscular dystrophy (DMD), enables the in vitro modeling of disease pathogenesis at a cellular level. By utilizing iPSCs, researchers can examine the effects of mutations on muscle development, function, and regeneration, as well as investigate the molecular pathways involved in muscle degeneration and repair. Moreover, iPSCs facilitate the study of oxidative stress, inflammation, and dysregulated signaling pathways that contribute to muscle dysfunction. The ability to generate large numbers of muscle cells from individual patients also offers the potential for personalized medicine, enabling the testing of novel therapeutics and optimizing treatment strategies tailored to each patient’s genetic profile. iPSCs are thus an invaluable tool in muscle physiopathology research, offering unprecedented insights into muscle diseases and facilitating the development of more effective therapies [29,31,32].

Collectively, the data presented in the papers published in the Special Issue “Skeletal Muscle Adaptations to Oxidative Stress” of the *International Journal of Molecular Sciences* offer significant new insights into the intricate processes of skeletal muscle adaptation to oxidative stress and the complex mechanisms underlying skeletal muscle regeneration in disease condition. A comprehensive understanding of these mechanisms is crucial for advancing our knowledge of muscle tissue physiopathology, particularly in the context of debilitating conditions that may lead to muscle degeneration and subsequent disability. In this regard, identifying novel strategies to enhance patient well-being and developing targeted therapeutic approaches is of paramount importance.

## Figures and Tables

**Figure 1:**
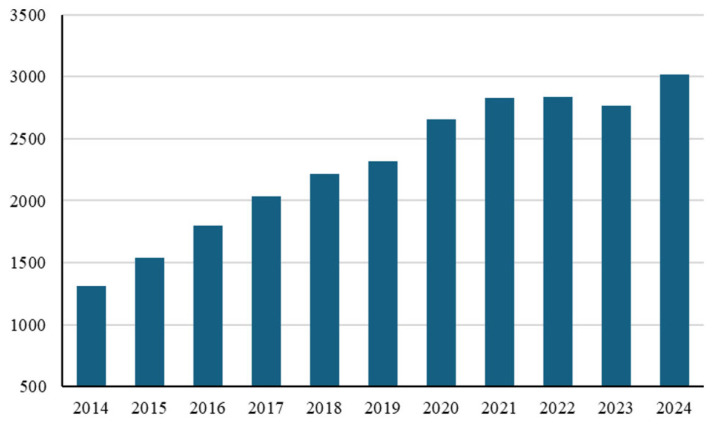
The number of publications indexed in PubMed from 2014 to 2024 applying the keyword “exercise” in combination with “cancer”.
